# Comprehensive Analysis of CD163 as a Prognostic Biomarker and Associated with Immune Infiltration in Glioblastoma Multiforme

**DOI:** 10.1155/2021/8357585

**Published:** 2021-08-05

**Authors:** Hao Li, Di Wang, Bolong Yi, Heng Cai, Zhuo Xi, Xin Lou, Zhen Li

**Affiliations:** ^1^Department of Neurosurgery, Shengjing Hospital of China Medical University, Shenyang, China; ^2^Liaoning Clinical Medical Research Center in Nervous System Disease, Shenyang, China; ^3^Key Laboratory of Neuro-Oncology in Liaoning Province, Shenyang, China

## Abstract

**Background:**

Glioblastoma multiforme (GBM) is the most common and aggressive primary malignancy in adults with high aggression. The prognosis of GBM patients is poor. There is a critical need for novel biomarkers for the prognosis and therapy of GBM.

**Methods:**

Differentially expressed genes (DEGs) in GBM were screened using TCGA cohort. Univariate and multivariate Cox regression analyses were performed on DEGs to identify the optimal prognosis-related genes. qRT-PCR was performed to verify the result.

**Results:**

A total of 5216 DEGs, including 2785 upregulated and 2458 downregulated genes, were obtained. Enrichment analysis revealed that these DEGs were mainly involved in the p53 signaling pathway and cell cycle, immune response, and MAPK signaling pathways. Moreover, the top 50 DEGs were associated with drug resistance or drug sensitivity. Prognosis analysis revealed that GBM patients with a high expression of CD163 and CHI3L2 had a poor overall survival, prognosis-free survival, and disease-specific survival. The univariate and multivariate analyses revealed that CD163 and age were independent factors affecting the prognosis of GBM patients. A validation study revealed that CD163 was upregulated in GBM tissues and associated with poor overall survival. Moreover, further analysis revealed that CD163 showed significant correlation with immune cells, immune biomarkers, chemokines, and chemokine receptors. We also identified several CD163-associated kinase, miRNA, and transcription factor targets in GBM, including LCK, miR-483, and ELF1.

**Conclusions:**

In conclusion, our study suggested CD163 as a prognostic biomarker and associated it with immune infiltration in GBM.

## 1. Introduction

Glioblastoma multiforme (GBM) is the most common and aggressive primary malignancy in adults with high aggression [1]. Until now, the etiology and pathogenesis of GBM are still far from clarified [2]. The standard treatment for GBM includes surgical tumor removal followed by ionizing radiation and alkylating chemotherapy [3]. However, there is no prognosis in the standard treatment for glioblastoma in the past two decades [4, 5]. The prognosis of glioblastoma patients is poor, with a median survival timeline of about 12 months, with a 5-year survival of about 10% [6]. These sobering data illustrate a critical need for novel biomarkers for the prognosis and therapy of GBM.

The immune microenvironment has been chronicled to exert a significant function in biological progress in cancer [7]. Immunotherapy based on immune checkpoint blockade is ever-increasingly suggested as the most promising therapy for GBM in addition to operative treatment, especially for patients with advanced GBM [8]. Though many immunotherapy methods, such as GBM vaccines, oncolytic viral therapies, immune-checkpoint suppressors, and chimeric antigen receptor T cell therapy, have been conducted in clinical trials, none of these have been applied for clinical treatment [7, 8]. Similarly, though some prognosis biomarkers, including MLK3 and P4HA1, have been identified in GBM at the genetic level, little of these have been applied for the prediction of the prognosis of patients [9–11]. Thus, it is necessary to identify novel biomarkers for the prognosis and therapy of GBM.

In recent years, with the development of sequencing technology and the establishment of various cancer databases, genomic research has become one of the most reliable means to accelerate the clinical and translational research and treatment of cancer. In our study, we aim to identify the prognosis biomarkers and therapy targets for GBM by mining databases. Our result may provide more suitable strategies to improve the anti-immune performance and prognosis prediction of GBM by using a high-throughput sequencing database.

## 2. Materials and Methods

### 2.1. Database and Gene Expression

Level 3 gene expression profiles (level 3 data) for GBM patients were isolated from The Cancer Genome Atlas (TCGA) data portal (https://tcga-data.nci.nih.gov/tcga/) and Chinese Glioma Genome Atlas (CGGA) data portal (http://www.cgga.org.cn/). The limma package (version: 3.40.2) of R software was used to explore the differential expression genes. The adjusted *p* value was analyzed to correct for false positive results in TCGA. “Adjusted *p* < 0.05 and log (fold change) > 2 or log (fold change) < −2” were defined as the thresholds for the screening of differential expression genes (DEGs).

#### 2.1.1. Heat Maps and Volcano Plots

Heat maps and volcano plots about DEGs were obtained using an R Project.

### 2.2. Enrichment Analysis

To further confirm the underlying function of potential targets, the data were analyzed by functional enrichment. Gene Ontology (GO) is a widely used tool for annotating genes with functions, especially molecular function (MF), biological pathways (BP), and cellular components (CC). Kyoto Encyclopedia of Genes and Genomes (KEGG) enrichment analysis is a practical resource for analytical study of gene functions and associated high-level genome functional information. To better understand the carcinogenesis of mRNA, the clusterProfiler package in R was employed to analyze the GO function of potential targets and enrich the KEGG pathway.

### 2.3. Drug Sensitivity Analysis

To analyze the correlation of DEGs and drug sensitivity, we collected 265 small molecules from the Genomics of Drug Sensitivity in Cancer (GDSC). We downloaded the area under the dose-response curve (AUC) values for drugs and gene expression profiles for all cancer cell lines. The Pearson correlation analysis was utilized to explore the correlation between DEGs and small molecules or drugs.

### 2.4. Survival Analysis

After separating the high/low expression group of GBM with the medium expression of DEGs, we used the Kaplan-Meier survival analysis to analyze the survival difference between these two groups. Log-rank tests were used to calculate *p* values and hazard ratio (HR) with 95% confidence interval (CI). Univariate and multivariate cox regression analyses were conducted to detect the proper terms to build the nomogram. The forest was used to show the *p* value, HR, and 95% CI of each variable through the “forestplot” R package. A nomogram was developed based on the results of a multivariate Cox proportional hazard analysis to predict the 1-year, 2-year, and 3-year overall survival. The nomogram provided a graphical representation of the factors, which can be used to calculate the risk of survival for an individual patient by the points associated with each risk factor through the “rms” R package. *p* < 0.05 was considered as statistically significant.

### 2.5. Genetic Mutation Landscape

The genetic mutation data was obtained from the TCGA database. The “maftools” package in R software was applied to analyze and visualize the genetic mutation landscape. A horizontal histogram showed that the genes have the higher mutation frequency in GBM patients.

### 2.6. Immune Infiltration Analysis

Spearman correlation analysis was performed to explore the relation between gene expression and immune cell infiltration and the expression of immune biomarkers as well as immune checkpoints in TIMER (https://cistrome.shinyapps.io/timer/) [12] and CIBERSORT (https://cibersortx.stanford.edu/) [13]. These gene markers of tumor-infiltrating immune cells included markers of CD8+ T cells, T cells (general), B cells, monocytes, TAMs, M1 macrophages, M2 macrophages, neutrophils, natural killer (NK) cells, dendritic cells (DCs), T-helper 1 (Th1) cells, T-helper 2 (Th2) cells, follicular helper T (Tfh) cells, T-helper 17 (Th17) cells, Tregs, and exhausted T cells [14]. A *p* value of less than 0.05 was considered statistically significant.

### 2.7. LinkedOmics

LinkedOmics (http://www.linkedomics.org/) is a bioinformatics platform for genomic analysis based on the TCGA dataset [15]. The “interpreter module” of LinkedOmics performs pathway and network analyses of CD163. Data from the LinkFinder results were signed and ranked, and GSEA was used to perform analyses of GO (CC, BP, and MF), KEGG pathways, kinase-target enrichment, miRNA-target enrichment, and transcription factor-target enrichment. The rank criterion was a *p* value < 0.05, and 500 simulations were performed.

### 2.8. GeneMANIA

GeneMANIA (http://www.genemania.org/) is a bioinformatics tool developed for protein-protein interaction (PPI) network analysis and for promoting understanding of the functional association data of target genes [16]. In order to better understand the function behind the CD163-associated kinase_LCK network, miR-483 network, and transcription factor ELF1 network, we submitted these genes to GeneMANIA to construct a PPI network.

### 2.9. Validation of the Expression and Prognosis Value

The immunohistochemistry staining of target genes in GBM tissues and normal tissues was obtained from The Human Protein Atlas (https://www.proteinatlas.org/), a bioinformatics tool aimed at mapping all the human proteins in cells, tissues, and organs using an integration of various omics technologies [17].

GBM and normal brain tissues (*n* = 52) were obtained from patients from the Shengjing Hospital of China Medical University. All patients provided informed consent. Each patient did not receive any treatment before operation. Total RNA of human tissues was extracted with a TRIzol reagent (Vazyme, Nanjing, China). The synthesis of cDNAs corresponding to the mRNAs of interest depended on PrimeScript RT-polymerase (Vazyme) and SYBR-Green Premix (Vazyme) with specific PCR primers (Sangon Biotech Co., Ltd., Shanghai, China). Glyceraldehyde-3-phosphate dehydrogenase was used as an internal control. The 2^−*ΔΔ*Ct^ method was used to calculate fold changes. Primer sequences were as follows: GAPDH, forward: GCACCGTCAAGGCTGAGAAC, reverse: TGGTGAAGACGCCAGTGGA, and CD163, forward: CTACGAACTTCAGCCAGAGTGCACCTCAT, reverse: GTCATAATGAACTTCAGCTATTGCACAC. The differences of the CD163 expression and the prognosis of CD163 in GBM were evaluated with Student's *t*-test and Kaplan-Meier analysis in GraphPad Prism 7 software (GraphPad, Inc., La Jolla, CA, USA).

## 3. Results

### 3.1. Identification of DEGs and Associated Functions in GBM

**Figure 1:**
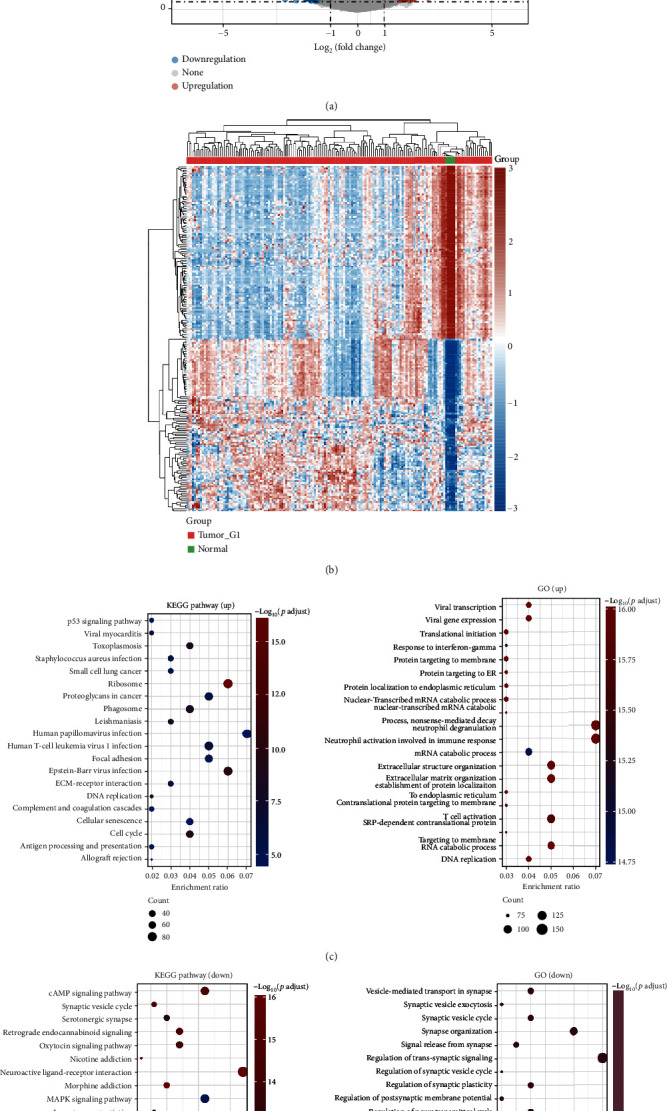
Differentially expressed genes in GBM and enrichment analysis. (a) Volcano plots showing differentially expressed genes in GBM with fold-change of 2 and a *p* value of 0.05. The red point in the plot represents the overexpressed mRNAs and the blue point indicates the downexpressed mRNAs with statistical significance. (b) Hierarchical clustering analysis of the top 50 differentially expressed genes in GBM. (c) The enriched KEGG signaling pathway and Gene Ontology (GO) analyses of upregulated genes. (d) The enriched KEGG signaling pathway and Gene Ontology (GO) analyses of downregulated genes.

The DEGs between GBM tissues and brain tissues were explored using TCGA GBM dataset. As a result, we obtained 5216 DEGs including 2785 upregulated and 2458 downregulated genes (Figure 1(a)).  Figure 1(b) shows the top 50 upregulated and downregulated genes. In order to explore the potential functions of DEGs in GBM, we performed enrichment analysis, including GO analysis and KEGG pathway analyses. As shown in Figure 1(c), these upregulated genes were mainly involved in the p53 signaling pathway and ribosome, proteoglycans in cancer, focal adhesion, DNA replication, and cell cycle in KEGG pathways. Moreover, GO analysis revealed that upregulated genes were mainly involved in vital transcription, vital gene expression, translational initiation, neutrophil activation involved in immune response, T cell activation, and DNA replication (Figure 1(c)). As for the result of downregulated genes, KEGG pathway analysis suggested that these genes were mainly involved in cAMP signaling pathways, the synaptic vesicle cycle, oxytocin signaling pathways, MAPK signaling pathways, and calcium signaling pathways (Figure 1(d)). Furthermore, GO analysis suggested that these downregulated genes were mainly involved in vesicle-mediated transport in synapse, synaptic vesicle exocytosis, signal release from synapse, regulation of neurotransmitter levels, potassium ion transport, cognition, and axonogenesis (Figure 1(d)).

### 3.2. Somatic Mutations in the GBM

**Figure 2:**
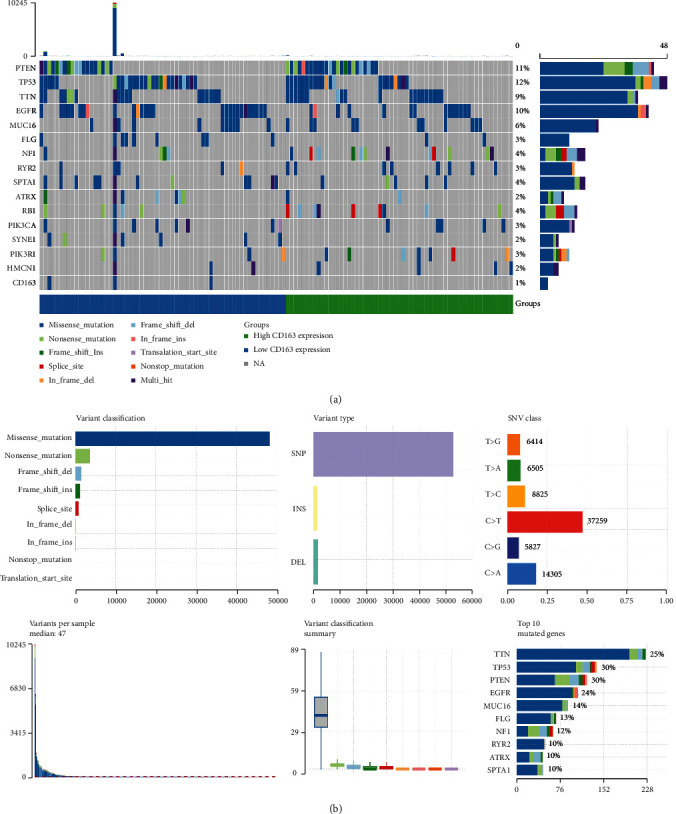
Genetic mutation landscape of differentially expressed genes in GBM. (a) Oncoplot displaying the somatic landscape of the GBM cohort. (b) Cohort summary plot displaying distribution of variants according to variant classification, type, and SNV class. Bottom part (from left to right) indicates mutation load for each sample and variant classification type. A stacked barplot shows top ten mutated genes.

To identify the somatic mutations of the patients with GBM in the TCGA database, we downloaded mutation data from TCGA and visualized using the “maftools” package in R software. A horizontal histogram showed that the genes have a higher mutation frequency in GBM patients, including TTN (25%), TP53 (30%), PTEN (30%), EGFR (24%), and MUC16 (14%) (Figures 2(a) and 2(b)). Missense mutations and nonsense mutations were the two most common types of mutation in GBM patients (Figures 2(a) and 2(b)). Scanning the variant types of mutations in GBM, single nucleotide polymorphism (SNP) was the most common type (Figure 2(b)). Moreover, C>T was the predominant mutation type in GBM (Figure 2(b)).

### 3.3. Drug Sensitivity Analysis of Top 50 DEGs in GBM

**Figure 3:**
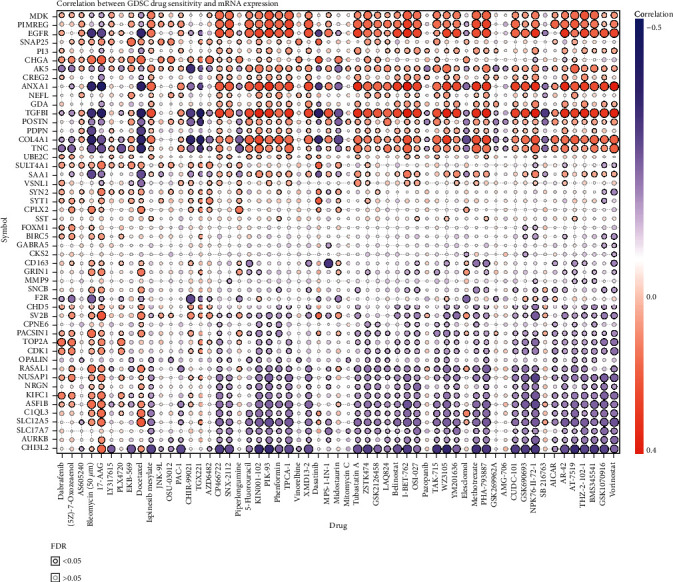
Drug sensitivity analysis of differentially expressed genes in GBM. The positive correlation means that the gene high expression is resistant to the drug and vice versa.

To develop cancer pharmacotherapy, a crucial way is to assess the link between DEGs and existing drug targets. In our study, we selected the top 50 DEGs (Supplementary Table 1) for further analysis and performed a drug sensitivity analysis. To explore the correlation of DEGs and drug sensitivity, the Pearson correlation coefficients of transcript levels and AUCs were used and normalized based on Fisher's *Z* transformation based on 265 small molecules from the Genomics of Drug Sensitivity in Cancer (GDSC), which was used before [18]. We observed that most of the top 50 DEGs show drug resistance (positive correlation) or drug sensitivity (negative correlation) (Figure 3).

### 3.4. Prognosis Value of Top 50 DEGs in GBM

**Figure 4:**
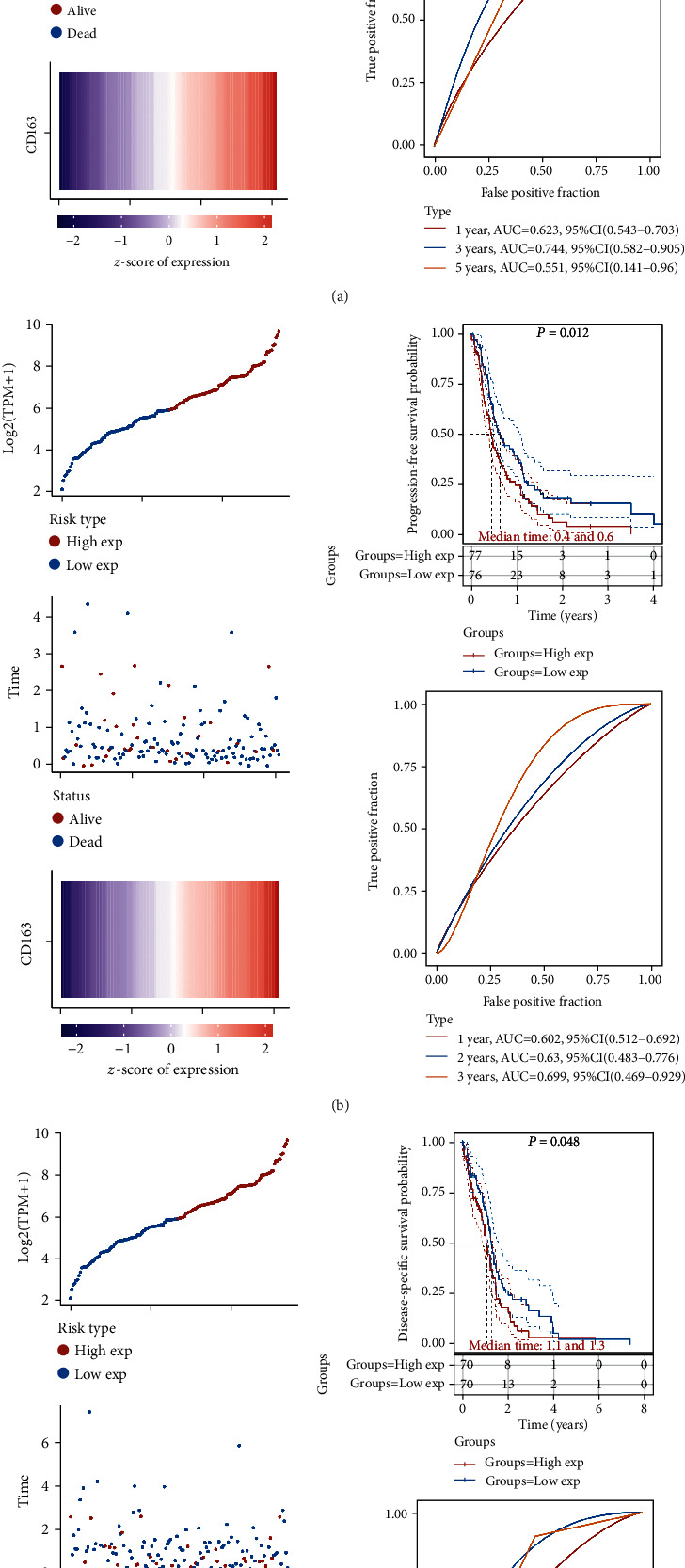
The prognosis analysis of differentially expressed genes in GBM in TCGA cohort. (a) Overall survival analysis of CD163 in GBM. (b) Progression-free survival analysis of CD163 in GBM. (c) Disease-specific survival analysis of CD163 in GBM.

**Figure 5:**
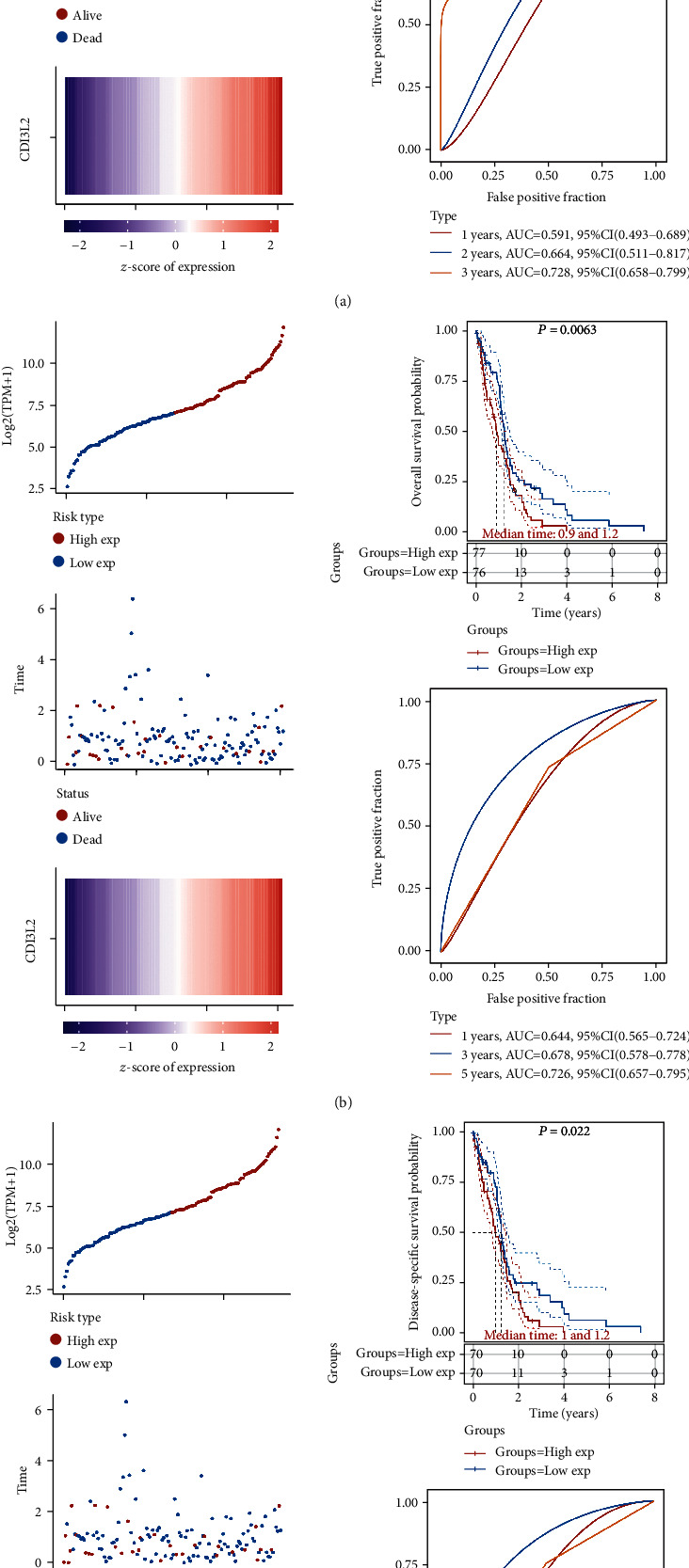
The prognosis analysis of differentially expressed genes in GBM in TCGA cohort. (a) Overall survival analysis of CHI3L2 in GBM. (b) Progression-free survival analysis of CHI3L2 in GBM. (c) Disease-specific survival analysis of CHI3L2 in GBM.

**Figure 6:**
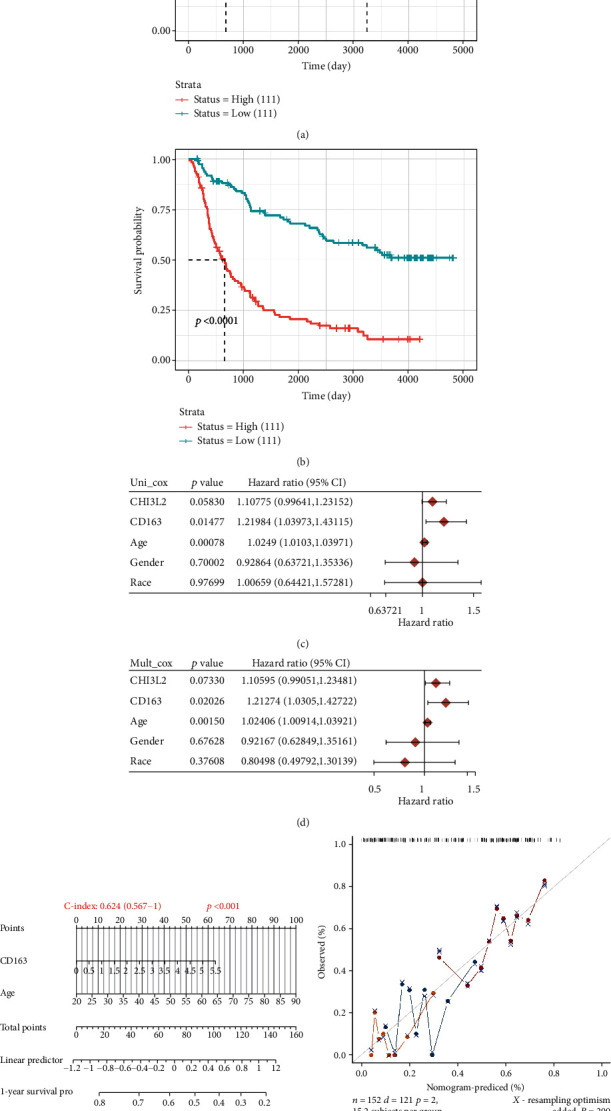
The prognosis analysis of differentially expressed genes in GBM in CGGA cohort. (a) Overall survival analysis of CD163 in GBM. (b) Overall survival analysis of CHI3L2 in GBM. (c, d) Hazard ratio and *p* value of constituents involved in univariate and multivariate Cox regressions considering clinical parameters and CD163 and CHI3L2. (e, f) Nomogram to predict the 1 y, 2 y, and 3 y overall survival of GBM patients. Calibration curve for the overall survival nomogram model in the discovery group. A dashed diagonal line represents the ideal nomogram, and the blue line, red line, and orange line represent the 1 y and 2 y observed nomograms.

We then performed prognosis value of top 50 DEGs in GBM, and the genes that were statistically significant in the overall survival, prognosis-free survival, and disease-specific survival are shown in Table 1. In overall survival, a total of 14 genes were associated with the prognosis of GBM patients. In prognosis-free survival, a total of 13 genes were associated with the prognosis of GBM patients. In disease-specific survival, a total of 11 genes were associated with the prognosis of GBM patients. Interestingly, the result revealed that GBM patients with a high expression of CD163 and CHI3L2 had a worse overall survival, prognosis-free survival, and disease-specific survival (Table 1, all *p* < 0.05). To be more specific, GBM patients with a high expression of CD163 had a poor overall survival (Figure 4(a), *p* = 0.0437, HR (95%CI) = 1.45(1.01 − 2.09)), prognosis-free survival (Figure 4(b), *p* = 0.012, HR (95%CI) = 1.60(1.11 − 2.30)), and disease-specific survival (Figure 4(c), *p* = 0.048, HR (95%CI) = 1.48(1.00 − 2.17)) with a 3-year AUC of 0.744 (Figure 4(a)), 0.63 (Figure 4(b)), and 0.749 (Figure 4(c)), respectively. And the risk score of each patient is shown in Figure 4. Moreover, GBM patients with a high expression of CHI3L2 had a poor overall survival (Figure 5(a), *p* = 0.0077, HR (95%CI) = 1.61(1.12 − 2.33)), prognosis-free survival (Figure 5(b), *p* = 0.0063, HR (95%CI) = 1.64(1.14 − 2.37)), and disease-specific survival (Figure 5(c), *p* = 0.022, HR (95%CI) = 1.58(1.07 − 2.35)) with a 3-year AUC of 0.664 (Figure 5(a)), 0.678 (Figure 5(b)), and 0.698 (Figure 5(c)), respectively. And the risk score of each patient is shown in Figure 5. In order to further verify our result, we then submitted CD163 and CHI3L2 to the CGGA cohort and performed a prognosis analysis. As expected, GBM patients with a high expression of CD163 (Figure 6(a), *p* < 0.0001) and CHI3L2 (Figure 6(b), *p* < 0.0001) had a poor prognosis in the CGGA cohort. These data demonstrated that CD163 and CHI3L2 might serve as prognostic biomarkers in GBM.

### 3.5. Building a Predictive Nomogram

We then resorted to a nomogram to construct a predictive model, considering clinicopathologic features and potential prognostic biomarkers, to construct a clinically applicable method that could predict the survival probability of the GBM patient. The univariate and multivariate analyses revealed that CD163 and age were independent factors affecting the prognosis of GBM patients (Figures 6(c) and 6(d)). We generated a nomogram to predict the 1-year, 2-year, and 3-year OS rates in the discovery group using the Cox regression algorithm (Figure 6(e)). The calibration plots for the 1-year and 3-year OS rates were predicted relatively well compared with an ideal model in the entire cohort (Figure 6(f)).

### 3.6. Validation of the Expression and Prognostic Value of CD163 in GBM

CD163 was selected for further study, and we performed validation research then. The immunohistochemistry staining from The Human Protein Atlas revealed that the immunohistochemistry staining of CD163 in GBM tissues and normal tissues was medium and not detected (Supplementary Figure 1A). Due to a similar role of CD8 and CD163 in immune infiltration, we also detected CD8 expression in GBM. The immunohistochemistry staining of CD8 in GBM tissues and normal tissues was medium and not detected (Supplementary Figure 1B). Moreover, qRT-PCR was performed to verify the expression and prognostic value of CD163 in GBM. As expected, CD163 expression was increased in GBM tissues (Supplementary Figure 2A, *p* = 1.2∗10^−6^). Further analysis suggested and GBM patients with a high CD163 level had a poor overall survival (Supplementary Figure 2B, *p* = 0.026) with an AUC of 0.723 in the ROC curve (Supplementary Figure 2C). These data further verified our result obtained above.

### 3.7. CD163 Were Associated with Immune Infiltration in GBM

**Figure 7:**
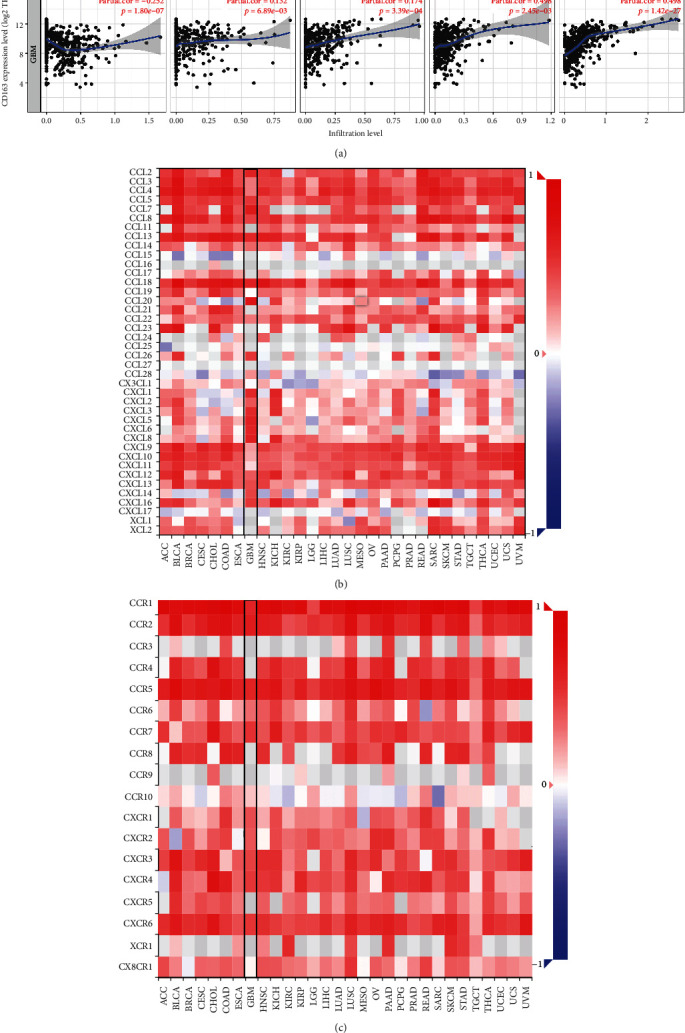
The association between CD163 and immune infiltration. (a) The association between CD163 expression and the abundance of B cells, CD8+ T cells, CD4+ T cells, macrophages, neutrophils, and dendritic cells. (b) The correlation between CD163 and the expression of chemokines in GBM. (c) The correlation between CD163 and the expression of chemokine receptors in GBM. ^∗^*p* < 0.05, ^∗∗^*p* < 0.01, and ^∗∗∗^*p* < 0.001.

Increasing evidences revealed that immune infiltration is an independent predictor of sentinel lymph node status and survival in cancers [14, 19, 20]. In order to explore the role of CD163 in GBM, we then detect the association between CD163 expression and immune infiltration in GBM. The result suggested that the CD163 expression was associated with the abundance of CD8+ T cells (Cor = −0.252, *p* = 1.80*E* − 7), CD4+ T cells (Cor = 0.132, *p* = 6.89*E* − 3), macrophage (Cor = 0.174, *p* = 3.39*E* − 4), neutrophils (Cor = 0.148, *p* = 2.45*E* − 3), and dendritic cells (Cor = 0.498, *p* = 1.42*E* − 27) (Figure 7(a)). We then verify this result using the CIBERSORT dataset, which revealed that the CD163 expression was associated with the abundance of neutrophils (Cor = 0.62, *p* = 1.38*E* − 17), macrophages (Cor = 0.64, *p* = 1.76*E* − 18), dendritic cells (Cor = 0.54, *p* = 7.41*E* − 54), and CD4+ T cells (Cor = 0.22, *p* = 0.006) (Supplementary Figure 3A).

Moreover, we also explore the association between CD163 expression and gene markers of tumor-infiltrating immune cells. As expected, the CD163 expression was positively correlated with most of gene markers of these tumor-infiltrating immune cells in both the TCGA and CGGA cohorts, including CD8A, CD8B, CD3D, CD3E, CD2, CD79A, CD86, CSF1R, CCL2, CD68, IL10, IRF5, COX2, VSIG4, MS4A4A, ITGAM, CCR7, KIR2DL4, HLA-DPB1, HLA-DQB1, HLA-DRA, HLA-DPA1, CD1C, NRP1, ITGAX, GATA3, STAT6, STAT5A, BCL6, STAT3, FOXP3, PDCD1, CTLA4, HAVCR2, and GZMB (Table 2). Immune checkpoints also play a vital role in immune infiltration of cancer [21]. In the current study, we found that CD163 expression increased as the expression of SIGLEC15, TIGFT, CD247, HAVCR2, PDCD1, CTLA4, and PDCD1LG2 increased (Supplementary Figure 3B, all *p* < 0.05). Chemokines and their receptors modulate immune surveillance in oncogenesis, metastasis, and response to immunotherapy [22]. Interestingly, the result demonstrated a strong correlation between CD163 and chemokines as well as chemokine receptors (Figures 7(b) and 7(c)). These evidences indicated the possible association between CD163 and immune infiltrates in GBM.

### 3.8. CD163-Associated Functions in GBM

**Figure 8:**
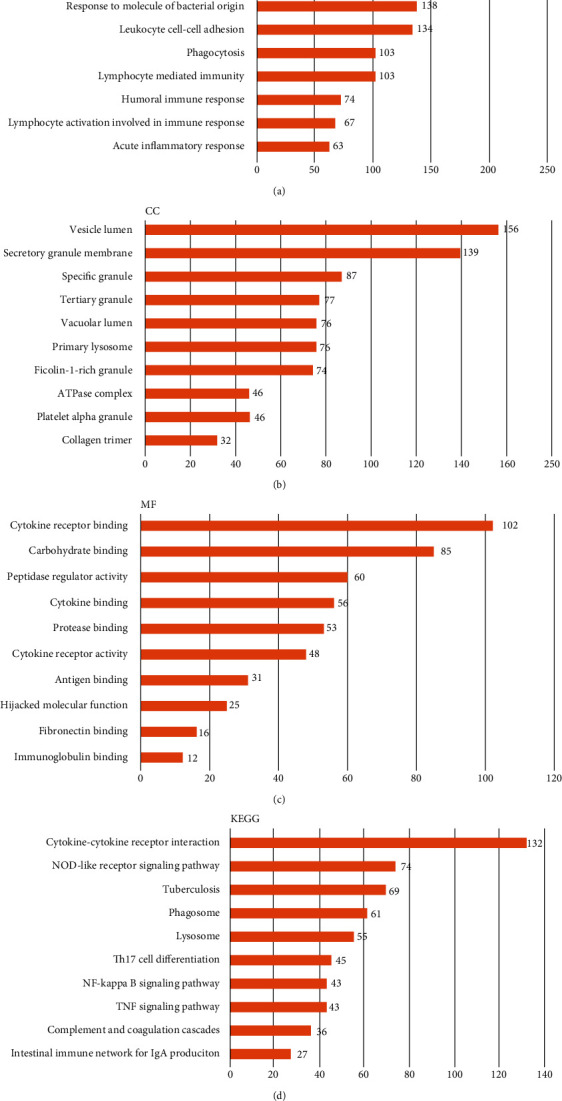
GO and KEGG pathway analyses of CD163 in GBM: (a) BP analysis; (b) CC analysis; (c) MF analysis; (d) KEGG pathway analysis.

In order to clarify the CD163-associated functions in GBM, we performed enrichment analysis using GSEA. The items in GO analysis are shown in Figure 8(a), revealing that CD163 were mainly involved in adaptive neutrophil-mediated immunity, immune response, leukocyte cell-cell adhesion, cytokine receptor binding, cytokine binding, and immunoglobulin binding. Furthermore, CD163 were mainly involved in cytokine-cytokine receptor interaction, NOD-like receptor signaling pathway, NF-kappa B signaling pathway, and TNF signaling pathway in KEGG pathway analysis (Figure 8(b)).

### 3.9. CD163-Associated Kinase, miRNA, or Transcription Factor Targets in GBM

**Figure 9:**
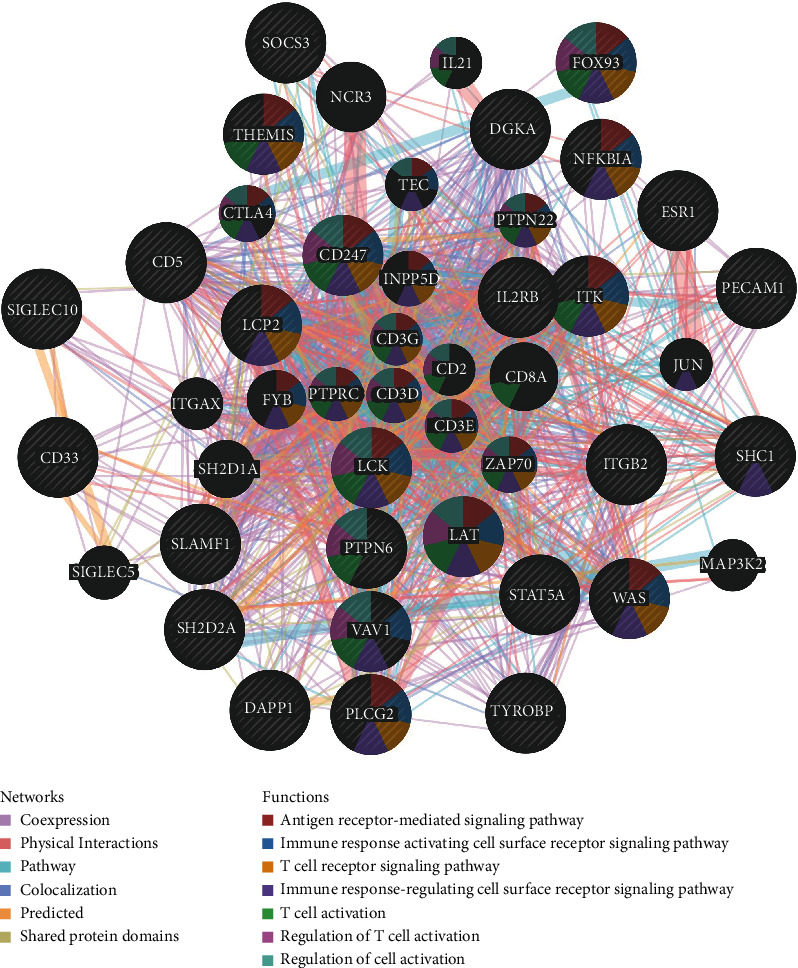
PPI network of LCK kinase-target networks. PPI network and functional analysis of the gene sets of LCK kinase-target networks. The different colors for the network nodes indicate the biological functions of the set of enrichment genes.

In order to further clarify the underlining mechanisms about how CD163 affected the tumorigenesis and progression of GBM, we finally explore CD163-associated kinase, miRNA, or transcription factor targets in GBM using GSEA in LinkedOmics. As a result, the result indicated that the top 5 most significant CD163-associated kinase targets in GBM were LCK, LYN, SYK, HCK, and ATR (Table 3, All *p* < 0.05). The PPI network based on the correlated genes of kinase LCK constructed by GeneMANIA indicated that kinase LCK was mainly related to the antigen receptor-mediated signaling pathway, immune response, and T cell receptor signaling pathway and activation (Figure 9). Moreover, the top 5 most significant CD163-associated miRNA targets in GBM were miR-483 (AGGAGTG), miR-485-5P (CAGCCTC), miR-197 (GTGGTGA), miR-499 (AGTCTTA), and miR-331 (CCAGGGG) (Table 3, All *p* < 0.05). The PPI network based on the correlated genes of miR-483 constructed by GeneMANIA indicated that miR-483 were mainly related to the regulation of lymphocyte activation, regulation of cell activation, and immune response (Supplementary Figure 4). The top 5 most significant CD163-associated transcription factor targets in GBM were V$ELF1_Q6, V$PEA3_Q6, V$E2F1_Q6, V$BACH2_01, and V$TEL2_Q6 (Table 3, All *p* < 0.05). The PPI network based on the correlated genes of ELF1 constructed by GeneMANIA indicated that ELF1 were mainly related to the regulation of transcription initiation from RNA polymerase II promoter, mediator complex, and nuclear hormone receptor binding (Supplementary Figure 5).

## 4. Discussion

The oncogenesis and progression of GBM is a complex multistep process, involved in a variety of gene expression patterns and other factors. Considering the heterogeneity and complex mechanism of GBM, clarifying the molecular mechanism of GBM is of significant importance for the therapy of GBM patients [23]. Moreover, the prognosis of GBM patients was poor. Though multidisciplinary comprehensive treatment, including surgery and chemo- and radiation therapy, had been used for GBM patients, the median survival time of GBM patients is only about 15 months [24]. And the five-year survival rate of GBM is about 0.05% to 4.7% [25]. Thus, it is quite necessary to explore new therapeutic targets and prognostic markers of GBM.

In order to explore new therapeutic targets and prognostic markers of GBM, we first identify the DEGs by comparing GBM tissues with normal tissues in the TCGA cohort. As a result, a total of 5216 DEGs including 2785 upregulated and 2458 downregulated genes were obtained. We then selected the top 50 DEGs for further analysis. In order to explore the potential of the 50 DEGs as the therapy targets of GBM patients, we detect the relation between DEGs and existing drug targets. Interestingly, we observed that most of the top 50 DEGs show drug resistance (positive correlation) or drug sensitivity (negative correlation). Therefore, these 50 DEGs had potential as the therapy targets of GBM patients, and further study should be performed to verify this result.

We explore the potential of the 50 DEGs as the prognostic biomarkers of GBM patients by performing prognosis analysis. And the data indicated that CD163 and CHI3L2 may serve as prognostic biomarkers in GBM and GBM patients with a high expression of CD163 and CHI3L2 which predicted a poor overall survival, prognosis-free survival, and disease-specific survival. These were consistent with a previous result, which found that CD163 predicts poor prognosis in glioma patients [26]. Actually, these CD163 and CHI3L2 have been suggested as prognostic biomarkers in other types of cancers. In oral squamous cell carcinoma, CD163 was a prognostic biomarker and associated with poor survival [27]. Another study suggested that high CD163 expression indicated a poor prognosis of patients with urothelial cell carcinoma [28]. In breast cancer, CD163 was related to postoperative radiotherapy and poor prognosis, indicating CD163 as a prognostic marker in breast cancer [29]. CHI3L2 was suggested as a prognostic biomarker for renal cell carcinoma, predicting high risk for postoperative progression [30]. Moreover, univariate and multivariate analyses were performed and demonstrated that CD163 and age were independent factors affecting the prognosis of GBM patients. And we select CD163 for further analysis.

Another important finding of our study is that CD163 was positively correlated with immune cells, immune biomarkers, chemokines, and chemokine receptors. We found that CD163 expression was associated with the abundance of CD8+ T cells, CD4+ T cells, macrophages, neutrophils, and dendritic cells. Moreover, CD163 expression was positively correlated with most gene markers of these tumor-infiltrating immune cells in both the TCGA and CGGA cohorts, including CD8A, CD8B, STAT6, STAT5A, and PDCD1. Actually, increasing evidences revealed that these immune cells and immune biomarkers exerted vital functions in tumor immune infiltration or served as a therapy target in GBM. The CD4+ T cell was linked to tumor angiogenesis and tumor progression in glioma patients [31]. Another study suggested that neutrophil-induced ferroptosis promotes tumor necrosis in the progression of GBM [31]. Moreover, monocytes could serve as a promising predictor for therapy responses of glioma patients [32]. Hung et al. suggested that PDCD1 and TIGIT dual checkpoint blockade enhances antitumor immunity and survival in GBM [33]. Moreover, STAT5A was a prognosis marker for GBM and involved in immune infiltration in GBM [34]. These evidences indicated that the possible association between CD163 and immune infiltrates in GBM and CD163 may serve as an immunotherapy target of GBM patients.

There is no doubt that our study had some limitations. Firstly, most analysis was performed at the mRNA level but not the protein level, and double immunohistochemistry staining should be performed to verify the protein expression of CD163 in GBM. Furthermore, it would be better to validate our results by performing in vivo and in vitro experiments.

In conclusion, our study suggested CD163 as a prognostic biomarker and associated it with immune infiltration in GBM.

## Figures and Tables

**Table 1:** Prognosis analysis top 50 differentially expressed genes in GBM.

Genes	Overall survival		Progression-free survival		Disease-specific survival	
	*p* value	HR (95% CI)	*p* value	HR (95% CI)	*p* value	HR (95% CI)
CD163	0.0437	1.45 (1.01-2.09)	0.012	1.60 (1.11-2.30)	0.048	1.48 (1.00-2.17)
CHI3L2	0.0077	1.61 (1.12-2.33)	0.0063	1.64 (1.14-2.37)	0.022	1.58 (1.07-2.35)
TOP2A	—	—	0.006	0.60 (0.42-0.86))	—	—
MMP9	—	—	0.044	1.45 (1.01-2.09)	—	—
NUSAP1	—	—	0.029	0.67 (0.47-0.96)	—	—
PIMREG	—	—	0.007	0.60 (0.42-0.87)	—	—
F2R	—	—	0.038	0.68 (0.47-0.98)	—	—
AURKB	—	—	0.026	0.66 (0.46-0.95)	—	—
PDPN	—	—	0.002	1.79 (1.24-2.59)	—	—
ANXA1	—	—	0.022	1.53 (1.06-2.21)	—	—
KIFC1	—	—	0.038	0.68 (0.48-0.98)	—	—
PI3	—	—	0.033	1.48 (1.03-2.13)	—	—
SAA1	0.033	1.49 (1.03-2.15)	0.042	1.46 (1.01-2.11)	—	—
TGFBI	0.019	1.55 (1.07-2.24)	—	—	0.016	1.62 (1.09-2.40)
PACSIN1	0.027	1.51 (1.05-2.18)	—	—	0.035	1.52 (1.03-2.26)
SULT4A1	0.023	1.52 (1.06-2.19)	—	—	0.025	1.56 (1.06-2.30)
VSNL1	0.012	1.59 (1.11-2.27)	—	—	0.039	1.50 (1.02-2.20)
SV2B	0.006	1.66 (1.15-2.39)	—	—	0.018	1.60 (1.08-2.37)
CHGA	0.016	1.57 (1.09-2.27)	—	—	0.033	1.54 (1.04-2.28)
SNCB	0.010	1.61 (1.12-2.32)	—	—	0.039	1.51 (1.02-2.22)
SLC12A5	0.005	1.70 (1.18-2.46)	—	—	0.005	1.76 (1.19-2.62)
NRGN	0.035	1.48 (1.03-2.14)	—	—	0.099	1.39 (0.94-2.06)
CPLX2	0.039	1.48 (1.02-2.14)	—	—	—	—
CREG2	0.031	1.49 (1.04-2.15)	—	—	—	—

**Table 2:** Correlation analysis between CD163 and gene biomarkers of immune cells in GBM.

Immune cells	Gene markers	GBM			
		TCGA		CGGA	
		Cor	*p* value	Cor	*p* value
CD8+ T cell	CD8A	0.202	∗	0.441	∗∗∗
	CD8B	0.241	∗∗	0.516	∗∗∗
T cell (general)	CD3D	0.378	∗∗∗	0.55	∗∗∗
	CD3E	0.392	∗∗∗	0.628	∗∗∗
	CD2	0.422	∗∗∗	0.624	∗∗∗
B cell	CD19	0.097	0.231	0.321	∗∗∗
	CD79A	0.187	∗	0.32	∗∗∗
Monocyte	CD86	0.58	∗∗∗	0.673	∗∗∗
	CD115 (CSF1R)	0.617	∗∗∗	0.462	∗∗∗
TAM	CCL2	0.567	∗∗∗	0.652	∗∗∗
	CD68	0.677	∗∗∗	0.746	∗∗∗
	IL10	0.593	∗∗∗	0.662	∗∗∗
M1 macrophage	INOS (NOS2)	-0.037	0.649	0.206	∗∗
	IRF5	0.317	∗∗∗	0.498	∗∗∗
	COX2 (PTGS2)	0.558	∗∗∗	0.447	∗∗∗
M2 macrophage	VSIG4	0.798	∗∗∗	0.735	∗∗∗
	MS4A4A	0.835	∗∗∗	0.851	∗∗∗
Neutrophils	CD66b (CEACAM8)	0.002	0.977	—	—
	CD11b (ITGAM)	0.607	∗∗∗	0.613	∗∗∗
	CCR7	0.475	∗∗∗	0.497	∗∗∗
Natural killer cell	KIR2DL1	0.13	0.11	—	—
	KIR2DL3	0.009	0.911	—	—
	KIR2DL4	0.236	∗∗	0.346	∗∗∗
	KIR3DL1	0.021	0.799	—	—
	KIR3DL2	0.002	0.985	—	—
	KIR3DL3	0.105	0.195	—	—
	KIR2DS4	0.184	0.0228	—	—
Dendritic cell	HLA-DPB1	0.57	∗∗∗	0.666	∗∗∗
	HLA-DQB1	0.328	∗∗∗	0.424	∗∗∗
	HLA-DRA	0.573	∗∗∗	0.736	∗∗∗
	HLA-DPA1	0.467	∗∗∗	0.687	∗∗∗
	BDCA-1 (CD1C)	0.349	∗∗∗	0.413	∗∗∗
	BDCA-4 (NRP1)	0.585	∗∗∗	0.763	∗∗∗
	CD11c (ITGAX)	0.171	∗	0.385	∗∗∗
Th1	T-bet (TBX21)	0.031	0.707	0.233	∗∗∗
	STAT4	0.318	∗∗∗	0.017	0.794
	STAT1	-0.022	0.783	0.45	∗∗∗
	IFN-g (IFNG)	0.134	0.0977	—	—
	TNF-a (TNF)	0.135	0.0967	0.126	0.0595
Th2	GATA3	0.277	∗∗∗	0.273	∗∗∗
	STAT6	0.442	∗∗∗	0.651	∗∗∗
	STAT5A	0.399	∗∗∗	0.623	∗∗∗
	IL13	-0.169	∗	0.09	0.18
Tfh	BCL6	0.218	∗∗	0.295	∗∗∗
	IL21	-0.009	0.91	—	—
Th17	STAT3	0.248	∗∗	0.681	∗∗∗
	IL17A	-0.019	0.82	—	—
Treg	FOXP3	0.296	∗∗∗	0.263	∗∗∗
	CCR8	0.393	∗∗∗	—	—
	STAT5B	-0.103	0.204	-0.079	0.238
	TGFb (TGFB1)	0.427	∗∗∗	0.62	∗∗∗
T cell exhaustion	PD-1 (PDCD1)	0.354	∗∗∗	0.516	∗∗∗
	CTLA4	0.429	∗∗∗	0.362	∗∗∗
	LAG3	0.023	0.777	0.37	∗∗∗
	TIM-3 (HAVCR2)	0.442	∗∗∗	0.622	∗∗∗
	GZMB	0.332	∗∗∗	0.565	∗∗∗

^∗^*p* < 0.05, ^∗∗^*p* < 0.01, ^∗∗∗^*p* < 0.001. —: no data.

**Table 3:** The kinase and transcription factor-target networks of CD163 in GBM.

Enriched category	Gene set	LeadingEdgeNum	*p* value
Kinase target	Kinase_LCK	26	0
	Kinase_LYN	23	0
	Kinase_SYK	18	0
	Kinase_HCK	9	0
	Kinase_ATR	34	0
miRNA target	AGGAGTG, miR-483	24	0
	CAGCCTC, miR-485-5P	54	0
	GTGGTGA, miR-197	27	0
	AGTCTTA, miR-499	17	0
	CCAGGGG, miR-331	38	0
Transcription factor target	V$ELF1_Q6	20	0
	V$PEA3_Q6	10	0
	V$E2F1_Q6	98	0
	V$BACH2_01	73	0
	V$TEL2_Q6	62	0

## Data Availability

The analyzed datasets generated during the study are available from the corresponding author on reasonable request.

## Supplementary Materials

Supplementary Materials Supplementary Table 1 The top 50 different expression genes in GBM. Supplementary Figure 1: the protein expression of CD163 and CD8A in GBM and normal tissue (The Human Protein Atlas). (A) The immunohistochemistry staining of CD163 in GBM tissues and normal tissues. (B) The immunohistochemistry staining of CD8 in GBM tissues and normal tissues. Supplementary Figure 2: validation of the expression and prognostic value of CD163 in GBM. (A) The relative expression of CD163 in GBM tissues and normal tissues. (B) The overall survival in STAD patients with a high and low expression of CD163. (C) The ROC curve of CD163 in predicting the prognosis of GBM patients. Supplementary Figure 3: the association between CD163 and immune infiltration. (A) The association between CD163 expression and the abundance of CD4+ T cells, macrophages, neutrophils, and dendritic cells. (B) The correlation between CD163 and the expression of immune checkpoints in GBM. ^∗^*p* < 0.05, ^∗∗^*p* < 0.01, ^∗∗∗^*p* < 0.001. Supplementary Figure 4: PPI network of miR-483-target networks. PPI network and functional analysis of the gene sets of miR-483-target networks. The different colors for the network nodes indicate the biological functions of the set of enrichment genes. Supplementary Figure 5: PPI network of transcription factor target of ELF1 networks. PPI network and functional analysis of the gene sets of transcription factor target of ELF1-target networks. The different colors for the network nodes indicate the biological functions of the set of enrichment genes.
