# Supplementary material for: Comprehensive Analysis of CD163 as a Prognostic Biomarker and Associated with Immune Infiltration in Glioblastoma Multiforme
Source: Biomed Res Int. 2021 Aug 5;2021:8357585. doi: 10.1155/2021/8357585 (PMC8363458; doi:10.1155/2021/8357585)
Supplement: Supplementary Materials — Supplementary Table 1 The top 50 different expression genes in GBM. Supplementary Figure 1: the protein expression of CD163 and CD8A in GBM and normal tissue (The Human Protein Atlas). (A) The immunohistochemistry staining of CD163 in GBM tissues and normal tissues. (B) The immunohistochemistry staining of CD8 in GBM tissues and normal tissues. Supplementary Figure 2: validation of the expression and prognostic value of CD163 in GBM. (A) The relative expression of CD163 in GBM tissues and normal tissues. (B) The overall survival in STAD patients with a high and low expression of CD163. (C) The ROC curve of CD163 in predicting the prognosis of GBM patients. Supplementary Figure 3: the association between CD163 and immune infiltration. (A) The association between CD163 expression and the abundance of CD4+ T cells, macrophages, neutrophils, and dendritic cells. (B) The correlation between CD163 and the expression of immune checkpoints in GBM. ∗p < 0.05, ∗∗p < 0.01, ∗∗∗p < 0.001. Supplementary Figure 4: PPI network of miR-483-target networks. PPI network and functional analysis of the gene sets of miR-483-target networks. The different colors for the network nodes indicate the biological functions of the set of enrichment genes. Supplementary Figure 5: PPI network of transcription factor target of ELF1 networks. PPI network and functional analysis of the gene sets of transcription factor target of ELF1-target networks. The different colors for the network nodes indicate the biological functions of the set of enrichment genes. [file 8357585.f1.zip › Supplementary Fig 5.pdf]

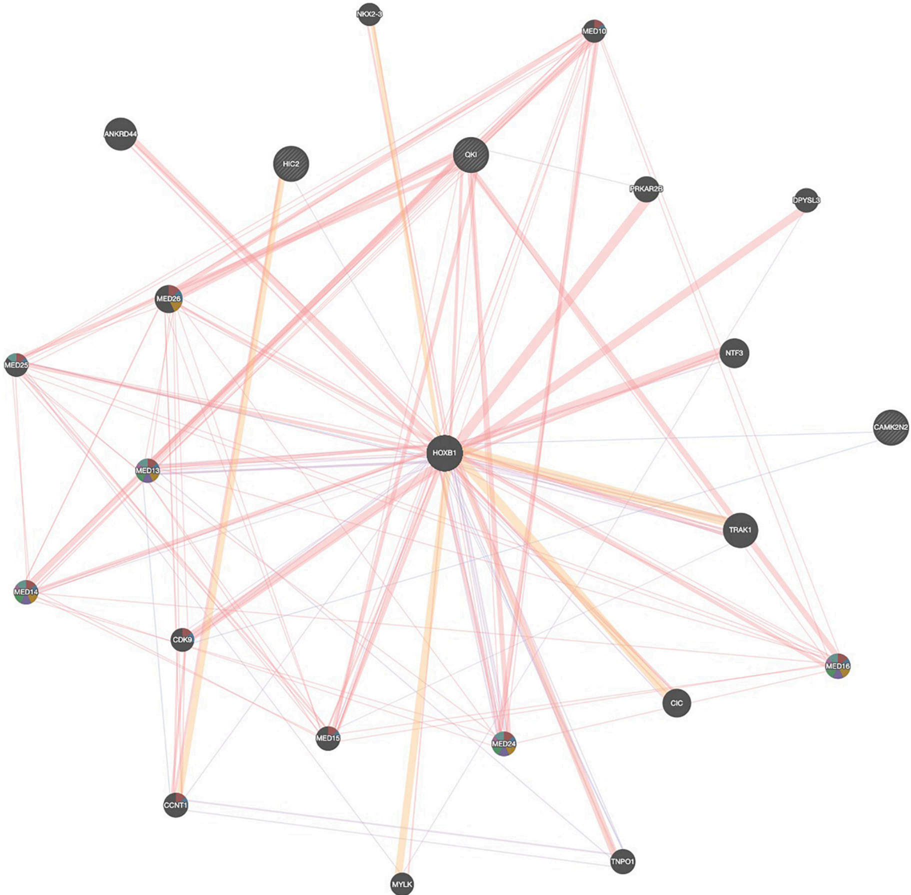

Networks

- Co-expression
- Physical Interactions
- Predicted
- Co-localization

Functions

- transcription initiation from RNA polymerase II promoter
- DNA-templated transcription, initiation
- mediator complex
- vitamin D receptor binding
- thyroid hormone receptor binding
- ligand-dependent nuclear receptor transcription coactivator activity
- nuclear hormone receptor binding
