# Supplementary material for: Comprehensive Analysis of CD163 as a Prognostic Biomarker and Associated with Immune Infiltration in Glioblastoma Multiforme
Source: Biomed Res Int. 2021 Aug 5;2021:8357585. doi: 10.1155/2021/8357585 (PMC8363458; doi:10.1155/2021/8357585)
Supplement: Supplementary Materials — Supplementary Table 1 The top 50 different expression genes in GBM. Supplementary Figure 1: the protein expression of CD163 and CD8A in GBM and normal tissue (The Human Protein Atlas). (A) The immunohistochemistry staining of CD163 in GBM tissues and normal tissues. (B) The immunohistochemistry staining of CD8 in GBM tissues and normal tissues. Supplementary Figure 2: validation of the expression and prognostic value of CD163 in GBM. (A) The relative expression of CD163 in GBM tissues and normal tissues. (B) The overall survival in STAD patients with a high and low expression of CD163. (C) The ROC curve of CD163 in predicting the prognosis of GBM patients. Supplementary Figure 3: the association between CD163 and immune infiltration. (A) The association between CD163 expression and the abundance of CD4+ T cells, macrophages, neutrophils, and dendritic cells. (B) The correlation between CD163 and the expression of immune checkpoints in GBM. ∗p < 0.05, ∗∗p < 0.01, ∗∗∗p < 0.001. Supplementary Figure 4: PPI network of miR-483-target networks. PPI network and functional analysis of the gene sets of miR-483-target networks. The different colors for the network nodes indicate the biological functions of the set of enrichment genes. Supplementary Figure 5: PPI network of transcription factor target of ELF1 networks. PPI network and functional analysis of the gene sets of transcription factor target of ELF1-target networks. The different colors for the network nodes indicate the biological functions of the set of enrichment genes. [file Supplementary Fig 4.pdf › Supplementary Fig 4.pdf]

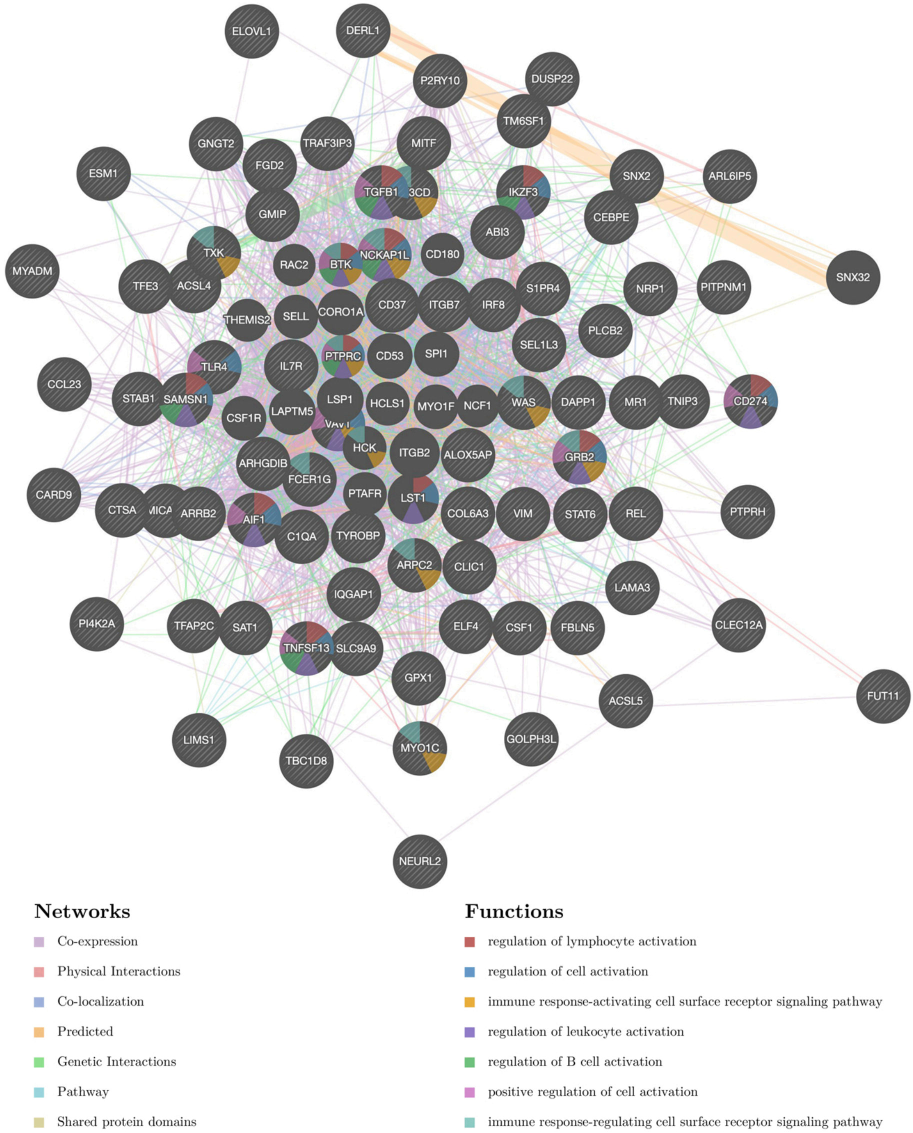
ELOVL1
DERL1
P2RY10
DUSP22
TM6SF1
GNGT2
TRAF3IP3
MITF
FGD2
ESM1
TGFB1
IKZF3
SNX2
ARL6IP5
GMIP
CEBPE
ABI3
TXK
MYADM
RAC2
BTK
NCKAP1L
CD180
SNX32
TFE3
ACSL4
S1PR4
NRP1
PITPNM1
THEMIS2
SELL
CORO1A
CD37
ITGB7
IRF8
PLCB2
SEL1L3
PTPRC
CD53
SPI1
TLR4
IL7R
CCL23
STAB1
SAMSN1
LSP1
HCLS1
MYO1F
NCF1
WAS
DAPP1
MR1
TNIP3
CD274
CSF1R
LAPTM5
HCK
ITGB2
ALOX5AP
GRB2
ARHGDIB
FCER1G
CARD9
CTSA
ARRB2
AIF1
PTAFR
LST1
COL6A3
VIM
STAT6
REL
PTPRH
C1QA
TYROBP
ARPC2
CLIC1
LAMA3
IQGAP1
PI4K2A
TFAP2C
SAT1
TNFSF13
SLC9A9
ELF4
CSF1
FBLN5
CLEC12A
GPX1
ACSL5
FUT11
LIMS1
TBC1D8
MYO1C
GOLPH3L
NEURL2
Networks
Co-expression
Physical Interactions
Co-localization
Predicted
Genetic Interactions
Pathway
Shared protein domains
Functions
regulation of lymphocyte activation
regulation of cell activation
immune response-activating cell surface receptor signaling pathway
regulation of leukocyte activation
regulation of B cell activation
positive regulation of cell activation
immune response-regulating cell surface receptor signaling pathway
